# Collaborative Research Priority Setting for Enhancing Primary Health Care Access Among the Nepalese Community in Canada: Community-Based Participatory Research

**DOI:** 10.3390/ijerph23040433

**Published:** 2026-03-30

**Authors:** Kalpana Thapa Bajgain, Mohammad Z. I. Chowdhury, Bishnu Bahadur Bajgain, Rudra Dahal, Kamala Adhikari Dahal, Nashit Chowdhury, Tanvir C. Turin

**Affiliations:** 1 Department of Community Health Sciences, Cumming School of Medicine, University of Calgary, Calgary, AB T2N 4N1, Canada; 2 Community Scholar and Citizen Researcher, Nepalese-Canadian Community, Calgary, AB T2N 1N4, Canada; 3 Department of Family Medicine, University of Calgary, Calgary, AB T2N 4N1, Canada; 4 Department of Population and Public Health, Alberta Health Services, Calgary, AB T2W 1S7, Canada

**Keywords:** priority setting, primary health care, enhancing, access, immigrants, Nepalese community, community engagement

## Abstract

**Highlights:**

**Public health relevance—How does this work relate to a public health issue?**

- Addresses inequities in primary health care access among immigrant populations, a persistent public health challenge in Canada.
- Centers immigrant community perspectives to better understand real-world barriers to accessing primary care services.

**Public health significance—Why is this work of significance to public health?**

- Provides population-based survey evidence on primary care access priorities within an immigrant community.
- Generates community-derived, gender-stratified priority data that can inform more responsive public health research agendas and service planning for immigrant populations.

**Public health implications—What are the key implications or messages for practitioners, policy makers and/or researchers in public health?**

- Public health practitioners and policymakers should incorporate community participation and gender-sensitive perspectives when designing primary care interventions for immigrant populations.
- Researchers should adopt transdisciplinary and collaborative priority-setting approaches to guide health system planning and improve access to care for diverse immigrant communities.

**Abstract:**

Background: Research concerning potential resolutions to immigrants’ health care access in Canada is limited, and the viewpoint of immigrant communities regarding priorities and feasible solutions remains inadequately captured. The objective of this article is to portray a research endeavor in which grassroots community members assumed the role of priority-setters for research on primary care access concerns. Aim: This cross-sectional study aimed to identify community-prioritized primary care access research topics among Nepalese Canadian immigrants in Calgary by ranking ten predefined issues based on perceived importance. Methods: We conducted community-based participatory research (CBPR) with the Nepalese community members in Canada. Participants were recruited using snowball sampling through community networks and rated topics using a 5-point Likert scale. A self-administered survey was used to collect participants’ rankings of ten predefined primary care access challenge themes. The themes were identified through comprehensive literature reviews undertaken by the research program team. The questionnaire was pilot-tested and refined based on feedback from team members before being administered. Results: A total of 401 Nepalese immigrants completed the survey, with 50.4% self-identifying as men. Among survey participants, significant gender differences were observed in sociodemographic characteristics, including age distribution, educational attainment, extended health insurance coverage, household income, and length of stay in Canada. Overall, health care cost and lack of resources were identified as the highest research priorities. While both men and women ranked these issues highly, women assigned greater priority to transportation- and culture-related barriers, whereas men generally assigned lower priority to these issues. Conclusions: There is a growing recognition that health solution priority-setting approaches should embrace transdisciplinary collaboration, with community participation as a pivotal factor. The results underscore the value of transdisciplinary, collaborative priority-setting approaches that center community participation to inform health research and interventions aligned with the needs of immigrant communities.

## 1. Background

In Canada, Primary Health Care serves as the initial point of entry for accessing health care services. This encompasses the prevention and treatment of common diseases and injuries, basic emergency services, coordination with or referral to other levels of care (such as hospitals and specialized care), primary mental health care, palliative and end-of-life care, health promotion, support for child development, primary maternity care, and rehabilitation services [1]. Challenges in accessing these services can lead to delays in seeking and receiving treatment, lack of awareness about preventive health services, diagnostic delays, increased financial strain on the health care system, and prolonged hospital stays [2,3].

Canada is renowned for its open immigration policies and is accepting immigrants from all over the world. As per the 2025 Annual Report, Canada received over 483,640 immigrants in 2024, and international migration accounted for vast majority of population growth in 2024 [4]. The 2026–2028 immigration level plan projects approximately 380,000 immigrants per year [4]. Despite the fact that many immigrants arrive in good health, their health status starts to deteriorate over the course of 10 years spent in Canada [5]. It has been reported that several elements consist of barriers related to financial hardship, cultural diversity, language proficiency, communication, societal influence, health system, structural barriers, and racism and discrimination, which avert immigrants from accessing health care in Canada [2,3]. To enhance and promote the quality of care for everyone, governments worldwide are continually interested in acquiring and evaluating the patient experience data to evaluate these barriers and factors [6].

This study focused on the Nepalese immigrant community in Canada to address unmet needs and primary care access issues with immigrant communities residing in Calgary, Canada. There has been a noticeable increase in the number of Nepalese immigrants to Canada. Nepalese immigrants to Canada make up a visible minority, as stated in Article 3 of the Employment Equity Act of 1995 [7]. According to Statistics Canada 2021, there were 23,425 immigrants whose mother tongue is Nepali residing in Canada, with 5900 living in the Province of Alberta [8]. The Non-Resident Nepali Association (NRNA) Canada, in 2021, recently estimated that the total number of Nepalese Canadians, including first and second generations, is roughly 50,000 [9]. A collaborative approach was used to engage Nepalese community members in Canada as partners in setting research priorities, ensuring that the study addressed locally relevant health needs [10]. This approach fosters trust, supports equitable decision-making, and enhances the relevance, uptake, and potential impact of research finding [10]. To date, concrete and rare studies appear to focus exclusively on the priority ranking of primary health care access of the Nepali community in Canada. This study aims to solicit input from Nepalese Canadian immigrants in Calgary to rank ten preidentified primary care access topics based on their perceived importance for research centered on solutions.

## 2. Materials and Methods

**Community-engaged research approach:** We implemented Community-based participatory research (CBPR) in this study as it is considered a successful strategy for identifying and meeting the needs of the immigrant population [10]. This power-equalizing strategy involves the immigrant population as collaborators in the research process to address the complex health disparities affecting their communities [11]. Similarly, to improve the health and well-being of immigrant/racialized populations, the community engagement approach can be vital by investigating the problems, locating their underlying causes, and brainstorming potential solutions [10,11]. Many scholars have provided anecdotal evidence on CBPR markedly when conducted with immigrants as equal partners that promote community agency, empowerment, and adoption of research findings [12,13]. While seeking solutions, a community-centered approach appears to be underemphasized, and the service delivery side frequently uses a top-down approach [14].

The study followed a CBPR framework, including partnership building, co-design of the survey instrument with community members, data collection, co-analysis, and dissemination. Community members actively contributed to all phases, helping define priority topics, review data, interpret findings, and shape knowledge translation strategies. Throughout the engagement process, several discussions were held with members of the Nepalese Community Society of Calgary (NCSC) to collaboratively prioritize ten pre-identified primary care access concerns based on their relevance. Additionally, the research team included community scholars and citizen researchers from the Nepali community who were engaged throughout the study, including design, data collection, analysis, interpretation, and dissemination through community meetings, presentations, and knowledge-sharing activities. These contributors are included as first authors and co-authors on this manuscript. Their involvement ensured cultural relevance, strengthened trust, and enhanced the applicability of the research priorities for the community.

**Study setting:** This study was conducted in Calgary because it is a rapidly growing destination for Nepalese immigrants and has the second largest Nepalese population in Canada after Toronto. The Calgary-based research team’s ongoing engagement with this community facilitated trust and collaboration. To our knowledge, no prior research has examined primary health care priorities in this population locally. This study represents an initial, community-engaged investigation, with plans to expand to other regions across Canada.

**Study design:** A self-administered survey was conducted to assess the primary care access needs of Nepalese immigrants living in Calgary, Alberta, from January to June 2019. The survey participants were asked to rank ten preselected topics related to primary care access, such as Workplace-related barriers, travel/transportation barriers, perceived discrimination, and so on. The objective of the research was to identify the most important and relevant issues for this population and to guide future interventions and policies.

**Developing the survey:** The questionnaire containing study priorities was co-developed by a team that included community scholars and citizen researchers. A comprehensive synthesis of the existing evidence on primary care access was first conducted to identify potential study themes [15]. This process drew on prior systematic reviews conducted by the team, as well as the group’s ongoing work and engagement within the community. The preliminary list of themes was then co-developed through an iterative process involving team members, including community scholars and citizen researchers, who contributed their experiential knowledge to review, refine, and contextualize the themes. This collaborative approach ensured that the final set of themes reflected both the existing evidence base and community-informed priorities, resulting in ten primary themes (see Table 1 for the list). Participants were presented with ten themes as separate statements and asked to rate each theme individually using a 5-point Likert scale (1 = “Not important,” 5 = “Extremely important”), with one response allowed per theme. Each theme was treated as an independent category, and mean scores were calculated for each theme to determine relative priority. This approach allows participants to express the perceived importance of each theme while maintaining comparability across themes. The survey questionnaire was pilot-tested and refined by team members before being administered.

**Priority Setting Approach:** This study employed a priority-setting approach with community members to identify and rank research priorities related to primary care access. Pre-identified topics were used to guide data collection; however, the primary aim was to systematically elicit community member rankings to inform research planning and resource allocation. This approach differs from conventional needs assessments, which primarily focus on identifying gaps or unmet needs, by emphasizing structured prioritization of topics based on community input, consistent with established priority-setting frameworks [14,16].

**Study population and data collection**: Individuals were eligible for this study if they were 18 years and older Nepalese immigrants living across Calgary, could read and write in English, and were willing to participate in the study. The NCSC and other local Nepalese communities were consulted before beginning the recruitment process. Participants were contacted through email, social media, and word of mouth. As they completed the enrolling process, the initial participants were urged to share study details with further contacts depending on their personal social networks.

Potential participants were informed about the research purpose and their roles. The self-administered questionnaires and the informed consent form were distributed to the interested and eligible participants. Participants returned the completed survey with the informed consent form after completion. A follow-up note was sent to non-respondent participants after two weeks. Finally, we received 401 completed surveys. Ethical approval for this study was obtained from the Conjoint Health Research Ethics Board, University of Calgary (REB 15-2325).

## 3. Analysis

The descriptive statistical analysis was performed on the categorical data and presented in frequencies and percentages. To retrieve the ranking of preferred research themes, the survey asked the respondents to choose the lowest priority to the highest priority (5-point Likert scale, ranging from lowest priority to highest priority) with the statement “**Workplace-related barriers**” (e.g., conflict of clinic visits with a time of work, loss of work hours and pay, fear of losing a job, avoiding injury reporting). We then offered ten statements about the ranking and asked respondents’ opinions using the same Likert scale. We analyzed responses to each statement asked on a Likert scale using summary statistics, including the mean (95% CIs) and the number (percentage) of responses. While Likert-scale responses are ordinal in nature, treating them as approximately interval-level data for calculating means is a common and accepted practice, particularly in large samples and when aggregating group-level responses [17,18]. Mean-based ranking was then used to aggregate ordinal ratings. This approach provides a clear, transparent, and reproducible method for capturing collective interest-holder priorities and is consistent with common practices in participatory research and research priority-setting exercises. For descriptive purposes, Likert responses were categorized as ≤2 = low priority, 3 = moderate priority, and ≥4 = high priority [19]. These thresholds were chosen to provide a clear summary of participant ratings and to highlight which themes were considered relatively more or less important by the community. This categorization is intended for interpretation and descriptive reporting only and does not imply formal statistical significance, consistent with common practices in research priority-setting studies using Likert scales.

Although participant gender was collected, this study was designed as a descriptive, community-driven prioritization exercise rather than a hypothesis-testing study. Consequently, formal statistical tests comparing priority rankings between men and women were not conducted. Instead, participant ratings were summarized and reported to describe patterns of community-identified priorities, consistent with standard practices in participatory research priority-setting exercises. Gender-stratified reporting was included purely to illustrate patterns and provide descriptive insight, as is common in participatory research, rather than to infer statistically significant differences.

All statistical analyses were performed using STATA, version 14.2.

## 4. Results

### 4.1. Participants’ General Characteristics

A total of 401 Nepalese immigrants residing in Calgary completed the survey. Of these, 202 (50.4%) self-identified as men and 199 (49.6%) as women. The demographic, socioeconomic, and health-related characteristics of participants overall and stratified by gender are presented in Table 2. Most participants were between 26 and 45 years of age (75.8%), with significant differences in age distribution by gender (*p* < 0.001). Majority of respondents were married (88.3%), and approximately half reported living in households with four or more members. Educational attainment differed significantly by gender, with a higher proportion of men reporting graduation-level education compared to women (49.0% vs. 24.8%, *p* < 0.001). Majority of participants were employed either full- or part-time (81.1%), although employment status differed marginally by gender (*p* = 0.058). Nearly all respondents reported having a regular family physician (95.8%), with no significant difference by gender. Extended health insurance coverage was reported by 74.6% of participants and was significantly more common among men than women (80.2% vs. 68.8%, *p* = 0.009). Household income distribution differed significantly by gender (*p* = 0.007), with women more likely to report annual household incomes of ≤$25,000. More than half of participants (55.0%) had lived in Canada for more than five years, although women were more likely than men to report a shorter length of stay (≤5 years; *p* = 0.007). Most participants rated their health status as very good or good (82.6%), and approximately one-third reported at least one chronic condition, with no statistically significant differences by gender.

### 4.2. Overall Ranking for Research Prioritization

The overall ranking of research priority topics is presented in Table 3. Across all participants, “**health care cost**” emerged as the highest-priority issue, with a mean score of 4.35 (95% CI: 4.25–4.45); more than four-fifths of respondents (81.6%; 95% CI: 77.4–85.1) rated this issue as a high priority (Likert score ≥ 4). “**Lack of resources**” was ranked second (mean score 4.03), with 69.6% (95% CI: 64.9–73.9) of participants identifying it as a high priority. Several topics received moderate priority ratings, including “**workplace-related barriers**”, “**cultural differences/preferences**”, and “**lack of continuity across providers**”, each with mean scores between 3.48 and 3.30. Approximately half of respondents rated workplace-related barriers (52.1%; 95% CI: 47.2–56.9) and cultural differences (49.6%; 95% CI: 44.7–54.5) as higher priorities. Relatively lower-ranked priorities included “**travel/transportation barrier**”, “**lack of knowledge**”, “**lack of interest from caregivers or the health system**”, and “**language barriers**”, with mean scores near or below the neutral midpoint of the Likert scale. “**Perceived discrimination**” was ranked lowest overall (mean score 2.72), with nearly half of participants (49.6%; 95% CI: 44.7–54.5) rating it as a low priority (Likert score ≤ 2).

### 4.3. Ranking for Research Prioritization by Gender (Men and Women)

Gender-stratified analyses revealed both shared and distinct patterns in research prioritization among men and women (Table 4 and Table 5). Among men, “**health care cost**” was the highest-ranked priority (mean score 4.26), with 78.2% (95% CI: 71.9–83.4) rating it as a high priority. “**Lack of resources**” ranked second (mean score 4.00). “**Workplace-related barriers**” and “**lack of continuity across providers**” were rated as moderate priorities. “**Perceived discrimination**” was the lowest-ranked issue among men, with over half (54.0%; 95% CI: 47.0–60.8) rating it as a low priority.

Among women, “**health care cost**” also ranked highest (mean score 4.44), with 84.9% (95% CI: 79.2–89.3) rating it as a high priority. “**Lack of resources**” was the second highest-ranked topic, followed by “**cultural differences/preferences**” and **“workplace-related barrier**”, both of which received higher mean scores and a greater proportion of high-priority ratings among women. As observed among men, “**perceived discrimination**” received the lowest overall priority rating among women, although a smaller proportion rated it as a low priority compared to men. Overall, while both men and women identified health care cost and lack of resources as top research priorities, women tended to assign greater importance to culture-related and transportation- barriers, whereas men generally assigned relatively lower priority to these issues.

## 5. Discussion

This study explores the community-identified priorities for primary care access among Nepalese Canadian immigrants residing in Calgary. In all, there were notable gender-based differences in age distribution, level of education, household income, and length of stay in Canada. Health care cost and lack of resources emerged as the top research priorities. Although both men and women rated these concerns as highly important, women placed greater emphasis on transportation and culture-related barriers, while men tended to view these issues as less critical.

In an ideal context, engaging end users in health research priority-setting generates a stronger evidence base to guide policies and interventions. However, there is a paucity of public/patient engagement in the research [16,20]. The literature has often mentioned different obstacles for not pursuing more community engagement, including community members not being distinguished as capable individuals, structural issues such as failure of communication, dearth of socially engaged groups, organizational issues, cultural issues, and conflict of interest [21,22]. The present study was conducted with the collaboration of grassroots community members for priority setting of health research.

The ranking of the topics in order of the research priorities identified the following top priorities: “health care cost”, “lack of resources”, “workplace-related barrier”, “cultural differences/preferences/perception”, and “travel/transportation barrier”. Consistent with the result of this study, the finding from the study by Turin et al. probing the prioritization of primary health care issues showed that lack of resources, health care cost, workplace-related barriers, and lack of knowledge were top priorities [14]. These findings are also consistent with a broader body of Canadian immigrant health literature, which has identified financial constraints, transportation challenges, system navigation difficulties, and limited awareness of available services as key barriers to accessing primary care across diverse immigrant groups [2,3,15]. This consistency suggests that the priorities identified in this study reflect systemic challenges within the health care system. This aligns with the assessment of European best practices that determined that formal structural barriers, lack of awareness regarding health care rights, and financial constraints predominantly impede overarching access to care for vulnerable migrants [23]. At the same time, the relatively higher prioritization of cultural differences and transportation barriers among women in this study adds nuance to existing evidence by highlighting how gender may shape the lived experience of these barriers within specific communities [3,24].

Participants perceived a “lack of continuity across providers”, “lack of knowledge,” and lack of interest in caregiver/system provider as moderate priorities, which could be dealt with in a secondary way, as the participants expressed that they have less impact on their health. On the other hand, language barriers and perceived discrimination were the lowest priorities among the participants. This lower prioritization of language barriers contrasts with findings from previous studies in immigrant populations, where language has often been identified as a major barrier [2,3,15]. This difference may reflect relatively higher levels of English proficiency or adaptation within this specific community, or the presence of informal support networks that help mitigate communication challenges. Furthermore, studies examining primary care access from systemic or provider perspectives tend to focus on addressing immediate clinical communication barriers [25], whereas immigrant communities often conceptualize access issues within broader, multifaceted socioeconomic constraints.

It is worth noting, though, that despite the priority ranking, the issues are interrelated. Lack of familiarity with the Canadian health care system is a dominant barrier where individuals are unaware of the type of services patients can access, unaware of Canada’s universal system, and free or subsidized services [26]. Additionally, long waiting times and unavailability of services will lead to underutilization of health services as immigrants are preoccupied with their daily financial requirements due to their socioeconomic status [27]. These all are interconnected to health care costs being a major barrier, as most immigrants, because of lack of Canadian work experience and their qualifications not being recognized, are placed in low-wage jobs and often have little to no discretionary income. Hence, they struggle to carry out dental and eye check-ups, cannot pay for medical equipment, and cannot afford medication [26].

In the current study, we were interested in finding out the priorities of male and female participants. Both participants felt that the health care cost, lack of resources, transportation barrier, and workplace-related barriers need to be dealt with as higher-ranked hindrances. This finding is in line with the study conducted among Bangladeshi immigrants in Canada [14]. Due to gender inequalities, immigrant women face distinctive and additional challenges in accessing health care services. This gap is caused by a variety of reasons, including sociodemographic characteristics and cultural perception/differences, and women are often expected to take care of their family members [14,28]. Consequently, immigrant women are more likely to have an unmet primary health care need and poorer health outcomes [29].

From a policy and service delivery perspective, the identified priorities highlight several actionable areas. The prominence of health care cost suggests a need to improve affordability of services not fully covered under the publicly funded system, including medications, dental care, and vision care. The importance of workplace-related barriers points to the potential value of flexible service delivery models, such as extended clinic hours or virtual care options, to accommodate working populations. Similarly, transportation-related challenges indicate the need for geographically accessible services and community-based care models. Addressing cultural differences and preferences further underscores the importance of culturally responsive care, including the use of culturally appropriate health information and community engagement strategies.

The key strength of this study is that we used a pragmatic approach and community engagement approach where the grassroots community was engaged to identify health research priorities. To ensure rigor and trustworthiness, we co-created the survey questionnaire with the community we were going to do the research with, pre-tested the questionnaire for clarity, and applied consistent scoring and data cleaning procedures. Community members were actively involved in survey administration, interpretation of results, and validation of priority rankings to ensure that findings accurately reflected community perspectives. Knowledge translation was facilitated through co-created reports, community presentations, and meetings with the Nepalese community to ensure results were understandable, relevant, and actionable. However, several limitations need to be considered. This study may be subject to selection bias due to the use of snowball sampling [30], which likely recruited socially connected participants who may know each other and share similar characteristics or perspectives. As a result, the observed priority rankings may be influenced by the perspectives of more engaged or socially connected members of the community. This should be considered when interpreting the findings, as the perspectives of individuals who are less socially connected or less engaged in community networks may be underrepresented. Consequently, the generalizability of the findings beyond the study sample may be limited. However, snowball sampling is commonly used in research involving immigrant and other hard-to-reach populations, where formal sampling frames are often unavailable and community networks can facilitate participant recruitment. As the advantages of non-probability sampling depend on the researchers’ practical knowledge and expertise and ease of establishing trust and rapport with the community members, our citizen researchers leveraged their rapport with the target population via extensive community engagement activities to overcome this bias. Additionally, there might have been social desirability response bias, as participants felt under pressure to choose what they prioritized. Although data were collected prior to the COVID-19 pandemic, the identified priorities reflect long-standing primary health care access challenges. Issues such as language barriers, long waiting times, transportation challenges, navigation of services, and access to timely primary care persist in the current health system, and in some cases have been further amplified by the pandemic.

## 6. Conclusions

There is a growing recognition that health solution priority-setting approaches should embrace interdisciplinarity and collaboration, with community participation as a pivotal factor. Such involvement enhances the health care system and fosters the creation of interventions that more effectively cater to the community’s needs. Engaging immigrant communities through collaborative, participatory approaches ensures primary care interventions are culturally appropriate and responsive to the local needs. Policy makers and practitioners should incorporate gender-sensitive strategies and leverage community networks to improve access, while researchers should use transdisciplinary and collaborative priority setting approaches to guide health system planning and resource allocation for diverse immigrant populations.

## Figures and Tables

**Table 1:** Priorities for Primary Health Care Access research topics by Nepalese Immigrants.

Preselected Topics	Description
Workplace related barriers	Conflict of clinic visits with a time of work, loss of work hours and pay, fear of losing job, avoiding injury reporting.
Travel/Transportation barriers	Long distance to access health care, parking cost and availability, difficulty in public transportation.
Perceived discrimination	Unapproachable nurse/staff at clinic/ Emergency department, ethnic preference at clinic, workplace discrimination while prioritizing health issues.
Lack of resources leading to long wait-times	Shortage of doctors, specialist services, urgent care clinics, after-hours services, which could have prevented the use of the emergency room for non-urgent care, lack of specialized diagnostic lab services leading to long wait times getting appointments for tests, long wait at doctor’s clinic even after having an appointment.
Lack of interest of caregiver/system/provider towards building provider–patient relationships	Doctor is not giving enough time and not explaining in proper way to patients, patient can discuss only one problem per visit.
Lack of knowledge about Canadian health care system and low awareness and engagement with services available	How system works, what services are available, immigrants’ right to make complaints, lack of participation in cancer screening, nutritional services, healthy aging, psychological services, child-youth wellness.
Lack of continuity across provider(s) as various providers and institutions often appear to work in isolation from one another	Lack of record and patient history sharing at walk-in clinic setups; lack of follow up from walk-in clinic doctor(s) lack of availability of the same doctor across different visits during maternity care.
Language barriers affecting effective conversation	Patients having difficulty explaining health issues, or understanding doctor’s advice; doctors having trouble understanding patient’s problems, problem identifying and navigating resources.
Health care cost	Lack of insurance coverage for drugs, eye, dental, and ambulance cost.
Cultural differences/preferences/perceptions among both immigrants and service provider	Gender of a physician, different cultural and rebellious beliefs, prejudice, preconceptions, culturally insensitive health care system, and programs, beliefs that discussing disease or psychological issues exhibits weakness.

**Table 2:** Demographic characteristics of participants.

Variables	Men (N = 202), n (%)	Women (N = 199), n (%)	*p*-Value
**Age**			
≤25 years	16 (7.92)	16 (8.04)	<0.001
26–35 years	53 (26.24)	94 (47.24)	
36–45 years	89 (44.06)	68 (34.17)	
46–55 years	36 (17.82)	13 (6.53)	
56–65 years	5 (2.48)	8 (4.02)	
≥66 years	3 (1.49)	0 (0.00)	
**Marital status**			
Married	179 (88.61)	175 (87.94)	0.383
Single	22 (10.89)	20 (10.05)	
Other (Separated/Divorced or Widowed)	1 (0.50)	4 (2.01)	
**Family size**			
≤3 members	96 (47.76)	108 (54.55)	0.175
≥4 members	105 (52.24)	90 (45.45)	
**Educational attainment**			
Graduation	99 (49.01)	49 (24.75)	<0.001
Undergraduate degree(s) and lower	103 (50.99)	149 (75.25)	
**Employment status**			
Employed (full-time and part-time)	173 (85.64)	152 (76.38)	0.058
Unemployed	18 (8.91)	31 (15.58)	
Student	11 (5.45)	16 (8.04)	
**Family physician attachment**			
Yes	192 (95.05)	192 (96.48)	0.476
No	10 (4.95)	7 (3.52)	
**Extended health Insurance Coverage**			
Yes	162 (80.20)	137 (68.84)	0.009
No	40 (19.80)	62 (31.16)	
**Yearly household income**			
≤$25,000	12 (6.19)	34 (17.53)	0.007
≥$26,000–50,000	55 (28.35)	49 (25.26)	
≥$51,000–$75,000	70 (36.08)	63 (32.47)	
≥$76,000	57 (29.38)	48 (24.74)	
**Length of stay in Canada**			
Less than or equal to 5 years	74 (38.14)	100 (51.81)	0.007
5+ years	120 (61.86)	93 (48.19)	
**Self-reported health status**			
Excellent	22 (11.22)	14 (7.22)	0.276
Very good and good	156 (79.59)	166 (85.57)	
Fair and poor	18 (9.18)	14 (7.22)	
**Chronic diseases**			
Yes	59 (29.21)	66 (33.17)	0.392
No	143 (70.79)	133 (66.83)	

NB. Not all participants responded to every question; therefore, totals are less than N = 401 for some variables (family size, educational attainment, yearly household income, length of stay in Canada, and self-reported health status). Percentages are calculated using available data for each variable.

**Table 3:** Overall ranking of the Topics for Research Prioritization.

Statements	Mean Score (95% CI)	No. (%; 95% CI) All Participants	
		Choosing ≥ 4 of 5 on Likert Scale	Choosing ≤ 2 of 5 on Likert Scale
**Health care cost**	4.35 (4.25–4.45)	327 (81.55; 77.43–85.06)	29 (7.23; 5.07–10.22)
**Lack of resources**	4.03 (3.93–4.13)	279 (69.58; 64.88–73.90)	32 (7.98; 5.69–11.08)
**Workplace related barrier**	3.48 (3.37–3.60)	209 (52.12; 47.21–56.99)	72 (17.96; 14.49–22.04)
**Cultural differences/preferences/perception**	3.46 (3.34–3.57)	199 (49.63; 44.74–54.52)	88 (21.95; 18.15–26.28)
**Lack of continuity across provider(s) as various providers and institutions often appear to work in isolation from one another**	3.30 (3.19–3.41)	187 (46.63; 41.78–51.55)	90 (22.44; 18.61–26.80)
**Travel/Transportation barrier**	3.22 (3.10–3.34)	182 (45.50; 40.66–50.42)	113 (28.25; 24.04–32.88)
**Lack of knowledge**	3.21 (3.09–3.33)	150 (37.41; 32.79–42.26)	126 (31.42; 27.05–36.15)
**Lack of interest of caregiver/system provider**	3.14 (3.02–3.27)	152 (37.91; 33.27–42.77)	143 (35.66; 31.11–40.49)
**Language barriers**	3.00 (2.85–3.15)	160 (39.90; 35.20–44.79)	175 (43.64; 38.85–48.56)
**Perceived discrimination**	2.72 (2.60–2.84)	103 (25.69; 21.63–30.20)	199 (49.63; 44.74–54.52)

NB. ≥4 represents “high priority” and ≤2 represents “low priority”.

**Table 4:** Ranking of the Topics for Research Prioritization by Men Participants.

Statements	Mean Score (95% CI)	No. (%; 95% CI) of Men	
		Choosing ≥ 4 of 5 on Likert Scale	Choosing ≤ 2 of 5 on Likert Scale
**Health care cost**	4.26 (4.12–4.40)	158 (78.22; 71.96–83.40)	13 (6.44; 3.76–10.80)
**Lack of resources**	4.00 (3.86–4.15)	140 (69.31; 62.57–75.31)	19 (9.41; 6.06–14.31)
**Workplace related barrier**	3.45 (3.28–3.62)	102 (50.50; 43.59–57.38)	39 (19.31; 14.41–25.38)
**Lack of continuity across provider(s) as various providers and institutions often appear to work in isolation from one another**	3.25 (3.10–3.41)	94 (46.53; 39.72–53.48)	51 (25.25; 19.71–31.73)
**Cultural differences/preferences/perception**	3.19 (3.04–3.35)	81 (40.10; 33.53–47.05)	54 (26.73; 21.05–33.30)
**Lack of knowledge**	3.16 (2.99–3.33)	71 (35.15; 28.84–42.02)	68 (33.66; 27.45–40.50)
**Lack of interest of caregiver/system provider**	3.14 (2.97–3.31)	80 (39.60; 33.06–46.55)	74 (36.63; 30.24–43.54)
**Travel/Transportation barrier**	3.03 (2.87–3.20)	78 (38.61; 32.11–45.55)	67 (33.17; 26.99–39.99)
**Language barriers**	2.91 (2.69–3.12)	74 (36.63; 30.24–43.54)	93 (46.04; 39.24–52.99)
**Perceived discrimination**	2.59 (2.43–2.76)	44 (21.78; 16.60–28.04)	109 (53.96; 47.01–60.76)

NB. ≥4 represents “high priority” and ≤2 represents “low priority”.

**Table 5:** Ranking of the Topics for Research Prioritization by Women Participants.

Statements	Mean Score (95% CI)	No. (%; 95% CI) of Women	
		Choosing ≥ 4 of 5 on Likert Scale	Choosing ≤ 2 of 5 on Likert Scale
**Health care cost**	4.44 (4.30–4.58)	169 (84.92; 79.22–89.28)	16 (8.04; 4.97–12.76)
**Lack of resources**	4.05 (3.91–4.19)	139 (69.85; 63.08–75.86)	13 (6.53; 3.82–10.96)
**Cultural differences/preferences/perception**	3.72 (3.56–3.89)	118 (59.30; 52.29–65.94)	34 (17.09; 12.44–23.00)
**Workplace related barrier**	3.52 (3.35–3.68)	107 (53.77; 46.77–60.62)	33 (16.58; 12.01–22.45)
**Travel/Transportation barrier**	3.41 (3.25–3.57)	104 (52.53; 45.52–59.43)	46 (23.23; 17.84–29.66)
**Lack of continuity across provider(s) as various providers and institutions often appear to work in isolation from one another**	3.35 (3.20–3.50)	93 (46.73; 39.87–53.73)	39 (19.60; 14.63–25.74)
**Lack of knowledge**	3.26 (3.09–3.42)	79 (39.70; 33.10–46.70)	58 (29.15; 23.22–35.88)
**Lack of interest of caregiver/system provider**	3.15 (2.97–3.33)	72 (36.18; 29.77–43.13)	69 (34.67; 28.35–41.59)
**Language barriers**	3.10 (2.89–3.31)	86 (43.22; 36.47–50.23)	82 (41.21; 34.54–48.21)
**Perceived discrimination**	2.85 (2.67–3.02)	59 (29.65; 23.68–36.40)	90 (45.23; 38.40–52.23)

NB. ≥4 represents “high priority” and ≤2 represents “low priority”.

## Data Availability

The data used in this study are available on aggregated form on reasonable request from the corresponding author.
